# Methionine Sulfoxide Speciation in Mouse Hippocampus Revealed by Global Proteomics Exhibits Age- and Alzheimer’s Disease-Dependent Changes Targeted to Mitochondrial and Glycolytic Pathways

**DOI:** 10.3390/ijms25126516

**Published:** 2024-06-13

**Authors:** Filipa Blasco Tavares Pereira Lopes, Daniela Schlatzer, Mengzhen Li, Serhan Yilmaz, Rihua Wang, Xin Qi, Marzieh Ayati, Mehmet Koyutürk, Mark R. Chance

**Affiliations:** 1 Center for Proteomics and Bioinformatics, Department of Nutrition, School of Medicine, Case Western Reserve University, Cleveland, OH 44106, USA; fxb99@case.edu (F.B.T.P.L.); dms73@case.edu (D.S.); mxk331@case.edu (M.K.); 2 Department of Computer and Data Sciences, Case School of Engineering, Case Western Reserve University, Cleveland, OH 44106, USA; mxl999@case.edu (M.L.); serhan.yilmaz@case.edu (S.Y.); 3 Center for Mitochondrial Diseases, Department of Physiology & Biophysics, School of Medicine, Case Western Reserve University, Cleveland, OH 44106, USA; rihhua.wang@case.edu (R.W.); xxq38@case.edu (X.Q.); 4 Department of Computer Science, University of Texas Rio Grande Valley, Edinburg, TX 78539, USA; marzieh.ayati@utrgv.edu

**Keywords:** methionine oxidation, MS*ox*, ROS, proteomics, 5XFAD, Alzheimer’s disease, mass spectrometry

## Abstract

Methionine oxidation to the sulfoxide form (MS*ox*) is a poorly understood post-translational modification of proteins associated with non-specific chemical oxidation from reactive oxygen species (ROS), whose chemistries are linked to various disease pathologies, including neurodegeneration. Emerging evidence shows MS*ox* site occupancy is, in some cases, under enzymatic regulatory control, mediating cellular signaling, including phosphorylation and/or calcium signaling, and raising questions as to the speciation and functional nature of MS*ox* across the proteome. The 5XFAD lineage of the C57BL/6 mouse has well-defined Alzheimer’s and aging states. Using this model, we analyzed age-, sex-, and disease-dependent MS*ox* speciation in the mouse hippocampus. In addition, we explored the chemical stability and statistical variance of oxidized peptide signals to understand the needed power for MS*ox*-based proteome studies. Our results identify mitochondrial and glycolytic pathway targets with increases in MS*ox* with age as well as neuroinflammatory targets accumulating MS*ox* with AD in proteome studies of the mouse hippocampus. Further, this paper establishes a foundation for reproducible and rigorous experimental MS*ox*-omics appropriate for novel target identification in biological discovery and for biomarker analysis in ROS and other oxidation-linked diseases.

## 1. Introduction

Oxidative stress has long been implicated as a central player in aging, cellular dysfunction, and disease progression [1,2,3,4], and reactive oxygen species (ROS) have traditionally been viewed as toxic byproducts of metabolism that cause non-specific damage to cells. ROS include oxygen ions [singlet oxygen, superoxide (O_2_−)] or oxygen-containing radicals [e.g., hydrogen peroxide (H_2_O_2_)] and related species [5,6]. The propensity of unregulated ROS to modify proteins and DNA can disrupt multiple cellular organelles and processes and disrupt normal physiology [5]. Multiple models of stress and disease across diverse species evidence deleterious ROS signaling [7,8]. Oxidative damage has also been linked to aging [2], as chemical changes to macromolecules will accumulate unless repaired or replaced. Thus, managing the production and consequences of cellular or environmentally generated ROS is critical to maintaining homeostasis in a range of organisms across evolution, including humans [5,9].

The oxidizing equivalents of ROS can modify many cellular targets, but in proteins, sulfur-containing residues—including methionine, which can be oxidized to methionine sulfoxide—are the most susceptible. Methionine oxidation is known to have broad effects on protein structure and function, as oxidation alters methionine’s hydrophobicity and steric bulk [6], which can unfold proteins and expose hydrophobic cores. These kinds of structural changes, if allowed to accumulate, can logically lead to changes in protein functions. Reversal of methionine oxidation can be accomplished by methionine sulfoxide reductases (MsR), which are seen in organisms from bacteria to humans. Oxidation of methionine generates a chiral center and the S and R stereoisomers are reduced by the enzymes MsrA and MsrB, respectively, both using thioredoxin-linked redox cycles [10,11]. This provides a reversible redox-based system to control MS*ox*-mediated changes in protein structure and activity in response to oxidative signaling. CaMKII (Calcium/Calmodulin-dependent Protein Kinase II) exemplifies this concept [12]. Beyond the canonical phosphorylation-based activation mechanisms, CaMKII’s activity is also modulated through the oxidation of specific methionine residues (Met 281 and 282) [13]. This links oxidation with a key control enzyme in cardiovascular and brain development and disease pathology. Actin, as well, has reversible modifications at Met 44 and Met 47, modulating cytoskeletal rearrangements in multiple vertebrate models relevant to cell growth and cancer under the regulation of the MICAL family of enzymes [14,15]. Thus, MS*ox* speciation is a novel regulatory control mechanism potentially operating to connect ROS to other signaling pathways.

Beyond these specific regulatory switches at a global level, methionine oxidation may act as a sensor of cellular stress [1,2,16,17,18,19,20,21,22,23], where protein-incorporated MS*ox* is accumulated, depleted, and regulated by the inducible system of Msr or related enzymes. The biological effects of Msr variation by knock-out or knock-in have showed lifespan variation in flies related to antioxidant defense, but results in mouse were equivocal, showing tissue-specific mouse functions affected [24]. Global proteomics studies to discover and validate both enzymatic and chemical sites of methionine oxidation speciation that would help inform such studies are scarce. Furthermore, quantification of methionine oxidation is considered challenging due to potential instabilities of the modifications during proteomics workups and in the handling and storage of samples. The development of reliable models and workflows to discover, verify, and validate MS*ox*-based regulatory phenomenon will assist connecting MS*ox* to ROS-mediated biology.

As examples, ROS- and oxidation-mediated effects are associated with both disease and aging across a range of models and species [1,2,24,25,26,27,28,29]. For example, Alzheimer’s disease and oxidation are strongly associated with evidence of lipid-, DNA-, and protein-based oxidation via glycation [30]. Further, amyloid peptides can mediate free radical reactions via Met 35 oxidation with enhanced neuronal toxicity [3,31,32]. Specific connections between AD and MS*ox* are suggested by decreased MsrA activity in the AD patient brain [33], while methionine sulfoxide reductase B2 (MsrB2) was inhibited in an AD mouse model. Further, compensation for the loss of MsrB2 in AD cell culture and animal models reverses AD-like pathology [34]. This argues that studies of MSox speciation are needed to better understand ROS-mediated oxidation-linked processes in AD.

Mouse models offer an excellent opportunity to study aging- and AD disease pathology-linked aspects of MS*ox* speciation. 5XFAD mice on a C57BL/6 background co-express five familial AD (FAD) mutations (amyloid precursor protein and presenilin 1 genes) that cause accumulation of amyloidogenic Aβ_42_, which spreads to cover most of the brain in parallel with astrocytosis and microgliosis by 4 months of age [35]. The mice later develop neuron loss around 9 months of age at the cortex and subiculum. Memory and cognitive impairments are observed in the mice as early as 4 months of age, which correlates with hippocampal synaptic dysfunction and age-dependent behavioral deficits. Metabolomics studies of this model revealed a dysregulation in brain-based glucose and lipid metabolism prior to AD pathogenesis [36]. Proteome studies in the brain revealed temporal and sex-linked variation in proteins related to neuroinflammation [37]. This model is ideal for our initial exploration of MS*ox* speciation.

**Figure 1:**
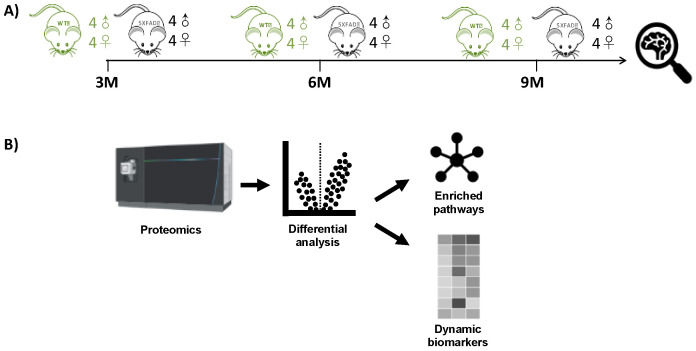
Methionine-oxidation characterization of 5XFAD and WT mouse models. (**A**) Longitudinal protein expression data from hippocampus tissues was extracted from Blasco et al., 2022 [37]. (**B**) Differential MS*ox* expression levels were extracted from raw LC-MS/MS data; from those signatures, both enriched pathways and candidate biomarkers were identified.

We examined proteome-wide levels of MSox in the 5XFAD mouse hippocampus at 3, 6, and 9 months for AD and C57BL/6 (wild type) mice, with replicates including a balanced design of males and females, as shown in Figure 1. This provides an examination of MS*ox* variation in the AD model from early Aβ_42_ deposition to full-blown neuroinflammation [37] and an examination of MS*ox* variation in C57BL/6 from and “end of youth” stage (month 3) through late middle age (month 9) [38]. Thus, this study is designed to achieve the following:

- Examine the early aging-, sex-, and disease-dependent changes in MS*ox* levels;
- Identify peptide and protein targets specifically sensitive and resistant to the oxidation of methionine;
- Validate key changes with peptide-based absolute quantification; and
- Explore the stability of key MS*ox* sites to handling and storage.

The results form a basis for a rigorous and reproducible MS*ox*-omics for biological discovery of novel links between oxidation and disease biology to drive the development of new therapeutic targets in contexts including neurodegeneration, cancer, and beyond.

## 2. Results

### 2.1. Experimental Design

Global MS*ox* proteome profiling of the mouse hippocampus was conducted across various time points representing AD hallmarks. The experimental design in Figure 1 involved the collection of hippocampus tissue from 16 mice at each point and then processing the samples immediately for comparisons at that month. At each time point, there were eight WT C57BL/6 mice and eight 5XFAD mice, comprising four male and four female biological replicates. Label-free LC-MS analysis was performed on each set of 16 samples without fractionation.

Statistical analysis in the parent study (Figure 1A) focused on WT versus 5XFAD comparisons, followed by sex-linked analysis at each of the three individual time points. Although processing all samples from a time point shortly after sacrifice in batches offered certain advantages, such as efficiency and sample stability, it posed challenges in comparing individual groups across time points due to difficulties in the absolute standardization of these different batches over time. Thus, in comparisons across time points, we focused on peptide and protein identification similarities and differences at each time point. With that caveat, we filtered peptides from the available data that featured methionine oxidation as a variable modification (Table S1). We the used these data to characterize the MS*ox*-ome, identify differentially expressed MS*ox*-proteins, and identify pathways possibly enriched in these signatures compared to the background mouse brain protein expression patterns.

### 2.2. Age-Dependent Increase in Global MSox

To characterize the MS*ox*-ome in our cohort, we used Peaks v10.0. This software takes raw LC-MS/MS files and provides peptide sequencing, protein identification, and quantification information. To extract specific MS*ox* data from our raw files, we included methionine oxidation as a variable modification as a PEAKS search parameter. We found 396 total MS*ox* peptides in the 3-month-old mice, both in WT and 5XFAD, 603 and 606 MS*ox* peptides in 6-month-old WT and 5XFAD mice, and 819 and 822 MS*ox* peptides in 9-month-old WT and 5XFAD mice, reaching a total hippocampal MS*ox* coverage of 1095 peptides (Table 1 and Table S1). Differences in the amount of MS*ox* proteins identified in 5XFAD compared to WT are negligible. Overall, the percentage of MS*ox* peptides as a function of the whole proteome increased over time from 3.59% to 4.48% (Table 1), showing that age, and not genetic background, dominates global MS*ox* changes in these mice.

### 2.3. Pathway Analysis Reveals Distinct Age-Dependent Signatures

**Figure 2:**
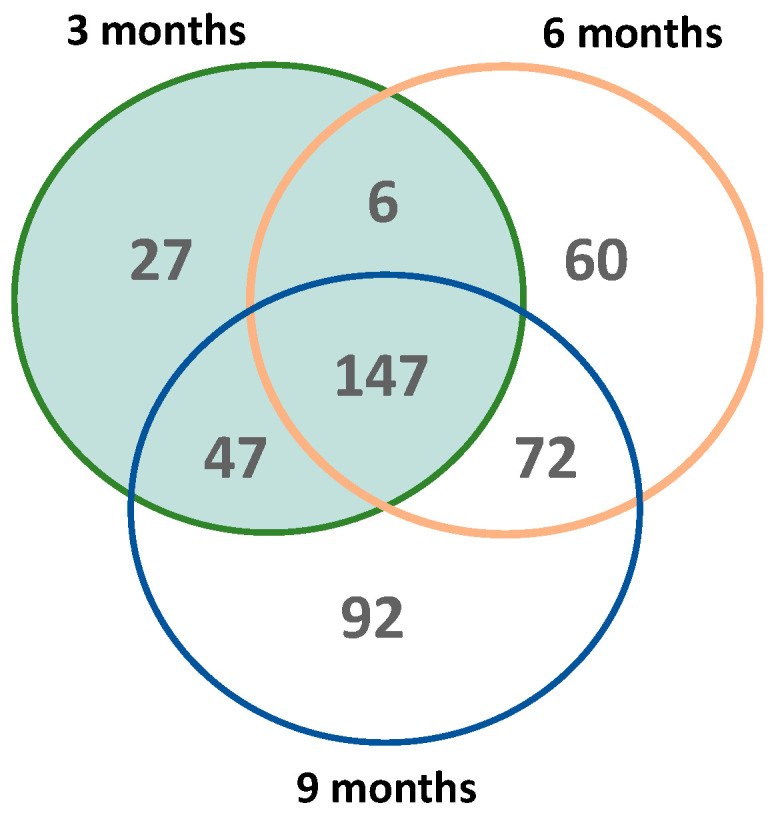
Global distribution of MS*ox* proteins across time in 5XFAD mice. The Venn diagram shows the number of shared M*Sox* proteins that are significantly expressed (5XFAD/WT 0.5 ≥ fold change ≥ 2 and *p* ≤ 0.1, unpaired *t*-test) between all timepoints (3 months—green; 6 months—orange; 9 months—blue).

To further characterize whether oxidation targets different protein-based methionine residues over time, we analyzed the overlap of each time-specific MS*ox*-ome. The Venn diagram highlights the temporal dynamics of the MS*ox*-ome in 5XFAD mice (Figure 2). Roughly a third of all MS*ox* proteins in this study were detected across all time points (Figure 2). Early time points (3 and 6 months) account for ~80% of the MS*ox*-ome (Figure 2). Moreover, 6%, 13%, and 20% of the MS*ox*-ome are time-specific expression signatures for 3, 6, and 9 months, respectively (Figure 2). Altogether, the data indicate that the MS*ox*-ome can be divided into two groups: constant oxidation targets (~33%) and dynamic targets (~66%).

To further understand whether these MS*ox* dynamics are associated with distinct biological processes, we performed separate enrichment pathway analysis using Reactome and cellular component perspectives to classify the MS*ox* proteins detected at the 3-, 6-, or 9-month time points. Indeed, we found different pathways to be enriched at different times and found those signatures to be distinct from whole proteome signatures (Table 2).

For example, at 3 months, no significant MS*ox* Reactome signatures were observed (at 10^−3^ or more). However, by six months, the Reactome pathway analysis revealed several significant enriched signatures, including glycolytic metabolism, glucose metabolism, and gluconeogenesis. Further L1CAM interactions, small-molecule transport, and chemical synapse transmission were seen to be significant at 6 months. At 9 months, the MS*ox* Reactome signatures also included innate immune systems and signal transduction as significant, while glucose-related metabolic themes were even more significant. For cellular component enrichments, L1 recycling and neurotransmitter release pathways along with glycolysis were significant for MS*ox*-annotated proteins at 6 months. Altogether, these data highlight that MS*ox* speciation accumulated in different proteins across coherent pathways across time.

Comparing the MS*ox*-ome with the whole-brain proteome-enriched pathways, we found that MS*ox*-enriched pathways are not an artifact of proteome coverage. Indeed, many pathways in bold show a significant enrichment exclusively in the MS*ox*-ome (Table 2). The proteome showed significance in all cellular components; this was expected as our MS approach is a reliable approach to generating global proteome coverage. However, the MS*ox*-ome showed a distinct cellular coverage, notably a lack of coverage in the cytoplasm, cytosol, and organelle (Table 2). On the other hand, the MS*ox*-ome retained coverage of the axon and myelin sheath (Table 2). Altogether, the data highlight the interest of characterizing the MS*ox*-ome as it provides biological insights beyond what the whole proteome provides.

### 2.4. High-Abundance Proteins Are Susceptible to MSox Accumulation

**Figure 3:**
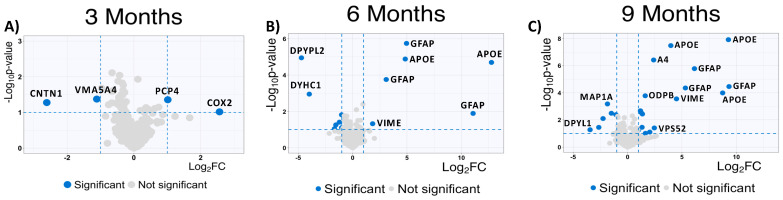
Global protein expression patterns highlight increase in upregulated methionine-oxidized peptides in AD. (**A**–**C**) Significantly upregulated methionine-oxidized peptides in AD mice; volcano plots highlight significantly dysregulated methionine-oxidized peptides in blue (5XFAD/WT 0.5 ≥ fold change ≥ 2 and *p* ≤ 0.1, unpaired *t*-test) (3 months, 6 months, and 9 months).

To complement our analysis of the MS*ox*-ome, we computed the fold changes of specific MSox peptides in WT mice compared to 5XFAD mice; these detailed quantitative comparisons at a specific time point are enabled by the experimental design of the parent study. We found some MS*ox* peptides to be differentially expressed in all time points (Figure 3A). Interestingly, starting at 6 months, the top upregulated peptides annotate to proteins known to be involved in neuroinflammation: GFAP, APOE, and VIME (Figure 3B,C).

At 9 Months, MSox peptides from GFAP (AEM[M]ELNDR, EQLAQQQVHVE[M]DVAKPDLTAALR and TQYEAVATSN[M]QETEEWYR), APOE (LGAD[M]EDLR, NEVT[M]LGSTEEIR and GWFEPIVED[M]HR), and VIME (E[M]EENFALEAANYQDTIGR) are significantly upregulated in 5XFAD (Figure 3). This is not surprising as these same peptides were found to be the most dysregulated in our whole-proteome analysis [37]. However, these results are associated with a pattern of MSox targeting highly expressed proteins (Figure 3).

**Figure 4:**
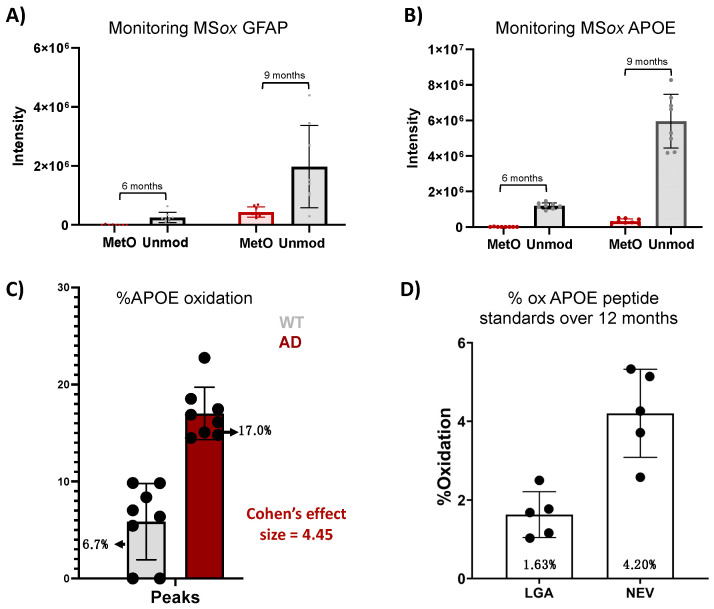
Analysis of methionine-oxidized APOE and GFAP in a mouse model of AD. (**A**,**B**) Tracking of AUC for unmodified and MS*ox* peptides in APOE (**A**) and GFAP (**B**) in 5XFAD mice; (**C**) Cohen’s effect size comparison between AUC (Peaks) measurements of methionine-oxidized APOE between WT and 5XFAD mice at 9 months; (**D**) bar graph compares the percentage of background oxidation in synthetic unmodified APOE peptides across 12 months, as measured by PRM.

To further explore the translational potential of the ox-APOE and ox-GFAP peptides as biomarkers, we need to clarify whether the differential expression of GFAP and APOE MS*ox* peptides is an artifact of the differential total protein expression (Figure 4). To do so, we looked at the area under the curve (AUC) summing APOE peptides LGAD[M]EDLR and NEVT[M]LGSTEEIR and GWFEPIVED[M]HR, GFAP peptides AEM[M]ELNDR, EQLAQQQVHVE[M]DVAKPDLTAALR, and TQYEAVATSN[M]QETEEWYR, and their total unoxidized peptide AUC values. No MS*ox* APOE nor MS*ox* GFAP were detected at 3 months. At 6 months, levels close to the lower limit of detection were registered for both MS*ox* APOE (NEVT[M]LGSTEEIR) and MS*ox* GFAP (AEM[M]ELNDR and EQLAQQQVHVE[M]DVAKPDLTAALR) in one sample (Figure 4A,B). At 9 months, there was an increase in the AUC of both unmodified GFAP and APOE by ~14% and~100%, and MS*ox* GFAP and APOE by ~73% and 51%, respectively (Figure 4A,B). To understand whether these increases in MS*ox* levels reflected changes in stoichiometry, we chose to focus our analysis on the APOE protein at 9 months (Figure 4C,D). Therefore, we compared the average MS*ox*-APOE% between WT (n = 8) and 5XFAD (n = 8) mice for the two APOE peptides above. For WT mice, an average of 6.7% APOE oxidation was seen, while for 5XFAD mice, an average of 17.1% oxidation for the two peptides was seen. These results reflect a 4.45 Cohen’s effect size among the two groups (Figure 4C). These data confirm a significant difference in MS*ox* APOE stoichiometry between WT and 5XFAD; thus, MS*ox* speciation targets APOE in this model. To further explore the ability to monitor MS*ox* APOE peptides as biomarkers, we tested whether targeted MS approaches would be adequate to monitor these peptides. The scientific community’s resistance towards MS*ox* being a biologically relevant PTM hinges in part on the fact that MS*ox* can also potentially occur or be removed spontaneously. However, the data here show very reproducible MS*ox* measurements, even on samples analyzed at separate times. Further, to assess whether parallel reaction monitoring (PRM) would be adequate to monitor MS*ox* APOE changes, we decided to track the spontaneous oxidation levels of unmodified APOE peptide standards (LGADMEDLR and NEVHTMLGQSTEEIR) over a 12-month period.

We developed a PRM quantitative assay specific to LGADMEDLR (Table S2) and NEVHTMLGQSTEEIR (Table S2) and their MS*ox* forms LGAD[M]EDLR (Table S2) and NEVHT[M]LGQSTEEIR (Table S2), for which we achieved linearity (R^2^ of >0.996) in all standard curves, demonstrating the rigor and reproducibility of this assay. Once linearity was established, we measured the oxidation in LGADMEDLR and NEVHTMLGQSTEEIR standards over time. We found average oxidation levels of 1.63% and 4.20%, respectively, with modest variability across the five samplings (Figure 4D, Table S2). Both levels of oxidation registered lower than the MS*ox* levels in both WT and 5XFAD mice (Figure 4C), indicating that PRM is a reliable method to monitor MS*ox* APOE peptides.

## 3. Discussion

**Figure 5:**
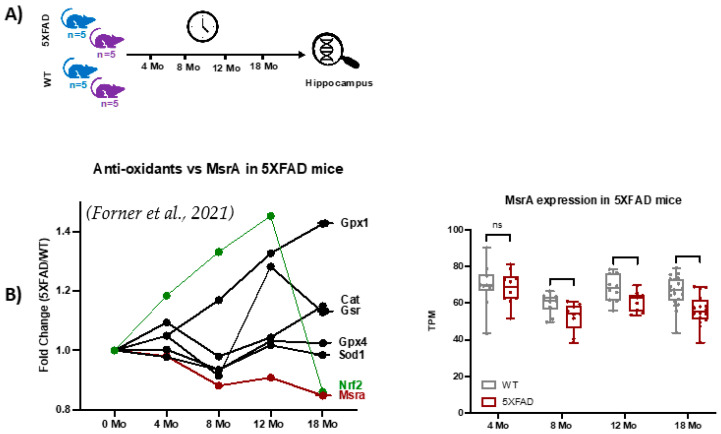
Functional genomics data for 5XFAD mice [39]. (**A**) Longitudinal RNAseq expression data from hippocampus tissues of 5XFAD mice at 4, 8, 12, and 18 months was extracted from [39]. (**B**) The plot shows longitudinal variations in MsrA, nuclear factor erythroid 2–related factor 2 (Nrf2) transcription factor, and antioxidant enzymes.

This study analyzed the global MS*ox* proteome of mouse hippocampus across various time points representative of Alzheimer’s disease (AD) hallmarks. To provide context for the functional genomics background of the 5XFAD model, we extracted selected gene expression data from a study like this proteomics study, with slightly different time points, as seen in Figure 5A, where the first time point of 4 months is near the 3-month point in proteomics and data up to 18 months are seen. Examination of the data showed strong evidence of ROS-related activation in the 5XFAD model vs. WT as Nrf2, a classic transcription factor mediating the gene expression of oxidation defense, is upregulated through 12 months (Figure 5B). Attendant oxidation defense proteins like glutathione peroxidases, catalase, and superoxide dismutase also show increased gene expression confirming the signaling landscape (Figure 5B). On the other hand, MsrA levels are trending lower with age and disease. These data drove our interest in specifically measuring MS*ox* speciation across the same time frames.

We identified a total of 1095 M*Sox* peptides in the WT and 5XFAD mouse hippocampus; however, not all M*Sox* peptides were identified across all time points. Indeed, by comparing MS*ox*-omes, we found an accumulation of M*Sox* peptides with the progression of time: 396, 606, and 822 at 3, 6, and 9 months, respectively (Figure 2). We found that only roughly a third of all MSox-annotated proteins are detected at all three time points (Figure 2), which highlights the temporal variation in MS*ox* targets in the mouse brain. Without further study, we cannot directly understand whether temporal effects are driven by accumulating MS*ox* vs. potentially decreased repair or how local speciation influences observed MS*ox*. However, pathway analysis at 3-, 6-, and 9-month intervals revealed transitions between glycolytic metabolism and small-molecule transport and chemical synapse transmission between the 6- and 9-month time points. Comparison with the whole proteome confirmed that MS*ox*-enriched pathways were not mere artifacts, highlighting their specific biological relevance. Furthermore, cellular component analysis revealed unique coverage patterns of the MS*ox*-ome, underscoring its importance for providing insights beyond conventional proteomic analyses.

With a goal to identify M*Sox* candidate biomarkers, we compared M*Sox* levels for selected peptides in WT and 5XFAD mice, which revealed a significant upregulation of selected peptides for GFAP, APOE, and VIME in 5XFAD mice starting at 6 months. This aligns with previous findings identifying these proteins as highly dysregulated in protein expression in 5XFAD. More detailed analysis of M*Sox* APOE showed a significant difference in M*Sox* APOE stoichiometry observed in 9-month mice for 5XFAD vs. WT. Parallel reaction monitoring (PRM) proved reliable for monitoring M*Sox* APOE peptides, as evidenced by low and relatively constant oxidation levels in peptide standards compared to mouse models (Figure 4C,D, Table S2). Our study underscores the potential clinical relevance of M*Sox* peptides in AD pathology and highlights the potential of PRM in clinical biomarker discovery for AD. The identification of APOE as a candidate MS*ox* biomarker is of great importance as it establishes yet another link between APOE and AD. Ever since GWAS studies identified APOE as the strongest AD genetic risk factor [40], a lot of effort has been made to elucidate the role of APOE in AD. Despite the abundance of knowledge, no APOE-based therapy has been effective to date. We hope future research may focus on exploring the functional consequences of M*Sox* APOE alterations and further validating PRM as a diagnostic tool for AD.

The characterization of MS*ox* APOE in 5XFAD could provide a novel angle to be explored in a therapeutical setting.

Future research may focus on exploring the functional consequences of M*Sox* alterations and further validating PRM as a diagnostic tool for AD.

## 4. Conclusions

Our findings contribute to a growing body of research on the role of senescence and protein oxidation in AD and connect this research to specific MSox speciation. We provide a benchmark for MS*ox*-ome information in mice across time, which includes preferentially targeted M*Sox* proteins and their associated biological pathways. Future research directions may involve investigating the functional implications of the identified MS*ox*-proteins (e.g., M*Sox* APOE), exploring potential therapeutic targets within enriched pathways, and further elucidating the interplay between senescence and protein oxidation in AD pathogenesis. Knock-out studies of Msr mouse models may also help identify specifically regulated M*Sox* sites, while longitudinal studies in human cohorts may provide insights into the development of diagnostic and therapeutic strategies for AD.

## 5. Materials and Methods

To characterize the temporal changes of MS*ox* expression levels in a mouse model of Alzheimer’s disease, we mined our previously published global proteome profiling data from 5XFAD [37].

### 5.1. Label-Free Quantitative Proteomic Analysis

Raw LC-MS/MS data were processed using Peaks v10.0 Software (Bioinformatics Solutions, Waterloo, ON, CAN) as described [41,42]. Peptide identification was performed within Peaks using UNIPROT database (UNIPROT_MOUSE_091219, # entries = 17,026). PEAKS search parameters were set to determine the following: mass error tolerance for precursor ions of 10 ppm, mass tolerance for fragment ions of 0.6 Da, trypsin enzyme specificity and included carbamidomethylation as a fixed variation plus methionine oxidation as a variable modification, and one missed cleavage. Label-free peptide identification was performed using default target decoy approach, which included PEAKS peptide score (−10logP) ≥ 15 and FDR threshold of 1%. Individual peptide abundance was determined by the area under the curve. All raw LC-MS/MS files and peptide abundance matrixes used in this article are publicly available at ProteomeXchange [43] and Consortium via the PRIDE [44] partner repository with the dataset identifier PXD030161 and can be found at https://doi.org/10.6019/PXD30161.

### 5.2. PRM

#### 5.2.1. Sample Preparation for PRM Assay

LGADMEDLR and NEVHTMLGQSTEEIR and their MSox forms LGAD[M]EDLR and NEVHT[M]LGQSTEEIR were synthesized (AQUA Basic-grade, >95% purity, Thermo Fisher Scientific, Waltham, MA, USA) and used as the standard for stability and linearity studies. Synthesized peptides were diluted in water to yield a final concentration of 1 pmol/μL for each standard peptide. Each standard was aliquoted and frozen at −80 °C. Aliquots were thawed for analysis at 0, 1, 2, 6, and 11 months. Dilutions were performed for each standard in 0.1% formic acid to yield a concentration of 50 fmoles/μL. Standard peptides were also diluted in 0.1% formic acid to yield concentrations of 0.1, 0.5, 1, 10, 25, 50, and 100 fmoles/μL for the linearity study.

#### 5.2.2. Development and Analytical Validation of Targeted MS Assays/Measurements

Both the stability and linearity study were analyzed by LC/MS using a Thermo Vanquish Ultra performance liquid chromatography system and an Orbitrap Exploris 480 mass spectrometer (Thermo, Waltham, MA, USA). The platform was operated in the positive nano-LC mode using Easy Spray source. The peptides were first desalted on a reversed-phase C18 trapping column (PepMap Neo Trap column Thermo, Waltham, MA, USA) by washing with 0.1% formic acid at 10 μL/min for 4 min. Subsequent chromatographic separation was performed using a reverse-phase C18 column (PepMap Neo 75 μm × 15 cm, Thermo, Waltham, MA, USA), and peptides were separated using a linear gradient of acetonitrile with 0.1% formic acid from 1% to 5% in 1 min followed by another linear gradient from 5% to 30% over a period of 29 min at a flow rate of 0.30 μL/min. A PRM experiment was employed to detect the standard peptides. The PRM approach was accomplished by specifying the parent mass of each peptide to be quantified for MS/MS fragmentation and then monitoring its fragment ions. AUC values were extracted for each peptide species using manual integration for stability and linearity analysis. The acquired data were processed and analyzed using Xcalibur 4.1.31.9 Quan Browser software (Thermo Fisher Scientific, Waltham, MA, USA). Each peptide was confirmed by comparing its retention time to the synthesized peptide. The % of oxidation for each peptide standard was calculated by following calculation: ((MS*ox* of standard/MS*ox* of standard + standard) × 100). Linear regression was performed for standard curves using Excel.

### 5.3. Enrichment Pathway Analysis

A PANTHER Overrepresentation Test with Reactome pathways annotation (Fischer’s exact test and FDR correction FDR *p* < 0.05) was performed on highly upregulated peptides in AD mice (5XFAD/WT Log2FC ≥ 4 and *p* ≤ 0.1, unpaired *t*-test) [45].

## Figures and Tables

**Table 1:** Global MS*ox* levels do not change with genetic background or sex. The tables summarize the total of detected methionine-oxidized peptides, MS*ox*-annotated proteins, and percentage of MS*ox*: (**A**) across time (3 months, 6 months, and 9 months) for both WT and 5XFAD mice; (**B**) in female and male 5XFAD mice.

**(A)**	**3 Months**		**6 Months**		**9 Months**	
	**WT**	**5XFAD**	**WT**	**5XFAD**	**WT**	**5XFAD**
MSox peptides	396	396	603	606	819	822
MSox proteins	228	228	282	285	358	358
MSox %	3.59	3.59	4.20	4.22	4.48	4.48
**(B)**	**3 Months**		**5XFAD Mice** **6 Months**		**9 Months**	
	**Female**	**Male**	**Female**	**Male**	**Female**	**Male**
MSox peptides	396	395	597	602	821	820
MSox proteins	228	228	283	284	358	357
MSox %	3.59	3.58	4.16	4.19	4.48	4.47

**Table 2:** **MS*ox* enrichments**. The table summarizes the enrichment pathway analysis FDR scores in **MS*ox*** (all methionine-oxidized proteins detected in each time point) and **Proteome** (all proteins detected in each time point) for both Reactome pathways and GO Cellular components. Bold values are more significant than 10^−3^.

		MSox			Proteome		
	Enriched Terms	3 M	6 M	9 M	3 M	6 M	9 M
**Reactome Pathways**	**Glucose metabolism**	1.76 × 10^−2^	**7.05 × 10^−5^**	**5.92 × 10^−5^**	1.00 × 10^0^	1.00 × 10^0^	5.56 × 10^−1^
	Immune system	1.05 × 10^−2^	3.90 × 10^−3^	**9.34 × 10^−5^**	4.28 × 10^−1^	**1.76 × 10^−5^**	**1.31 × 10^−11^**
	**Gluconeogenesis**	1.31 × 10^−2^	**3.32 × 10^−4^**	**1.22 × 10^−4^**	6.58 × 10^−1^	1.00 × 10^0^	1.00 × 10^0^
	**Organelle biogen and maintenance**	1.17 × 10^−2^	4.46 × 10^−3^	**4.07 × 10^−4^**	1.00 × 10^0^	2.99 × 10^−1^	7.69 × 10^−2^
	**Transport of small molecules**	ns	**6.46 × 10^−4^**	**4.29 × 10^−4^**	4.53 × 10^−1^	7.23 × 10^−1^	1.33 × 10^−1^
	**Transmission chemical synapses**	9.80 × 10^−3^	**5.43 × 10^−4^**	**6.07 × 10^−4^**	1.00 × 10^0^	7.11 × 10^−1^	2.47 × 10^−2^
	Innate immune system	ns	1.49 × 10^−2^	**8.32 × 10^−4^**	4.47 × 10^−1^	**9.58 × 10^−6^**	**1.25 × 10^−11^**
	Signal transduction	3.65 × 10^−2^	2.43 × 10^−2^	**8.37 × 10^−4^**	3.64 × 10^−1^	4.04 × 10^−1^	**2.89 × 10^−4^**
	L1CAM interactions	6.47 × 10^−3^	**6.29 × 10^−4^**	5.59 × 10^−3^	4.64 × 10^−2^	1.70 × 10^−1^	**1.14 × 10^−4^**
	**Recycling pathway of L1**	1.05 × 10^−2^	**6.32 × 10^−4^**	1.27 × 10^−3^	2.44 × 10^−1^	4.15 × 10^−1^	1.15 × 10^−3^
	**Glycolysis**	1.18 × 10^−2^	**7.92 × 10^−4^**	2.44 × 10^−3^	1.00 × 10^0^	1.00 × 10^0^	8.12 × 10^−1^
	**Neurotransmitter release cycle**	1.03 × 10^−2^	**8.38 × 10^−4^**	2.66 × 10^−3^	1.00 × 10^0^	ns	9.68 × 10^−2^
**Cellular Component**	**Cytoplasm**	ns	ns	ns	**1.41 × 10^−215^**	**1.49 × 10^−243^**	**9.63 × 10^−263^**
	**Intracellular anatomical structure**	ns	ns	ns	**3.51 × 10^−152^**	**1.19 × 10^−166^**	**6.39 × 10^−186^**
	**Cytosol**	ns	ns	ns	**8.87 × 10^−98^**	**4.40 × 10^−103^**	**4.91 × 10^−107^**
	**Organelle**	7.42 × 10^−3^	ns	5.02 × 10^−3^	**3.12 × 10^−113^**	**3.30 × 10^−124^**	**9.44 × 10^−128^**
	**Postsynapse**	3.53 × 10^−2^	5.53 × 10^−3^	6.98 × 10^−3^	**2.48 × 10^−112^**	**5.86 × 10^−117^**	**4.81 × 10^−112^**
	**Axon**	**3.05 × 10^−9^**	**2.80 × 10^−7^**	**4.35 × 10^−6^**	**5.43 × 10^−58^**	**2.93 × 10^−64^**	**2.52 × 10^−62^**
	**Myelin sheath**	**2.94 × 10^−29^**	**1.76 × 10^−30^**	**5.49 × 10^−30^**	**7.26 × 10^−79^**	**2.27 × 10^−72^**	**1.72 × 10^−68^**

## Data Availability

The mass spectrometry proteomics data relative to the original label free mass spectra have been deposited to the ProteomeXchange [42] Consortium via the PRIDE [43] partner repository with the dataset identifier PXD030161 and can be found at https://doi.org/10.6019/PXD30161.

## Supplementary Materials

The following supporting information can be downloaded at: https://www.mdpi.com/article/10.3390/ijms25126516/s1.
